# Hepatitis C Virus Phylogenetic Clustering Is Associated with the Social-Injecting Network in a Cohort of People Who Inject Drugs

**DOI:** 10.1371/journal.pone.0047335

**Published:** 2012-10-26

**Authors:** Rachel Sacks-Davis, Galina Daraganova, Campbell Aitken, Peter Higgs, Lilly Tracy, Scott Bowden, Rebecca Jenkinson, David Rolls, Philippa Pattison, Garry Robins, Jason Grebely, Alyssa Barry, Margaret Hellard

**Affiliations:** 1 Centre for Population Health, Burnet Institute, Melbourne, Victoria, Australia; 2 Department of Epidemiology and Preventive Medicine, Monash University, Melbourne, Victoria, Australia; 3 Centre for Research Excellence in Injecting Drug Use, Burnet Institute, Melbourne, Victoria, Australia; 4 Psychological Sciences, University of Melbourne, Parkville, Victoria, Australia; 5 The Kirby Institute for Infection and Immunity in Society, University of New South Wales, Darlinghurst, New South Wales, Australia; 6 Victorian Infectious Diseases Reference Laboratory, North Melbourne, Victoria, Australia; 7 Division of Infection and Immunity, Walter and Eliza Hall Institute, Parkville, Victoria, Australia; 8 Department of Medical Biology, University of Melbourne, Parkville, Victoria, Australia; University of Cincinnati College of Medicine, United States of America

## Abstract

It is hypothesized that social networks facilitate transmission of the *hepatitis C virus* (HCV). We tested for association between HCV phylogeny and reported injecting relationships using longitudinal data from a social network design study. People who inject drugs were recruited from street drug markets in Melbourne, Australia. Interviews and blood tests took place three monthly (during 2005–2008), with participants asked to nominate up to five injecting partners at each interview. The HCV core region of individual isolates was then sequenced and phylogenetic trees were constructed. Genetic clusters were identified using bootstrapping (cut-off: 70%). An adjusted Jaccard similarity coefficient was used to measure the association between the reported injecting relationships and relationships defined by clustering in the phylogenetic analysis (statistical significance assessed using the quadratic assignment procedure). 402 participants consented to participate; 244 HCV infections were observed in 238 individuals. 26 genetic clusters were identified, with 2–7 infections per cluster. Newly acquired infection (AOR = 2.03, 95% CI: 1.04–3.96, p = 0.037, and HCV genotype *3* (vs. genotype *1*, AOR = 2.72, 95% CI: 1.48–4.99) were independent predictors of being in a cluster. 54% of participants whose infections were part of a cluster in the phylogenetic analysis reported injecting with at least one other participant in that cluster during the study. Overall, 16% of participants who were infected at study entry and 40% of participants with newly acquired infections had molecular evidence of related infections with at least one injecting partner. Likely transmission clusters identified in phylogenetic analysis correlated with reported injecting relationships (adjusted Jaccard coefficient: 0.300; p<0.001). This is the first study to show that HCV phylogeny is associated with the injecting network, highlighting the importance of the injecting network in HCV transmission.

## Introduction

Globally, it is estimated that 170 million people are infected with the *hepatitis C virus* (HCV) [1]. The burden of disease associated with HCV is considerable. Approximately 75% of new HCV infections progress to chronicity and of those, 15–20% develop liver cirrhosis [2]. In developed countries, the main group at risk of HCV infection are people who inject drugs (PWID), with most new infections attributed to injecting drug use (Australia: 80%, USA: 65%) [3]. Globally, approximately ten million PWID are currently or previously infected with HCV and the estimated prevalence of HCV infection among PWID is 60% or greater in 37 countries [4], [5].

Social network epidemiology is a novel method that facilitates investigation of factors relating to the patterns connecting individuals socially [6]. Social network structure (the size, density, member position, and turnover of the network), composition (the socio-demographic traits of network members, the types of relationships between them, and their infection status), and behaviours (interactions between network members) have been linked to initiation, continuation, and cessation of injecting drug use, and sharing injecting equipment [7]. Moreover, the following characteristics of index participants' injecting partners have been shown to be important determinants of HCV infection: HCV status, age at first injecting [8], duration of injecting [9], geographic injecting location [10], the type of drugs used [11] and relationship with index participants [9], [11]. However, whilst social network characteristics have been linked to HCV infection status, this association may be due to confounding relationships between injecting risk behaviors and social network factors.

Molecular phylogenetics is the study of evolutionary relatedness among genetic sequences and can be used to reconstruct the shared history of sampled viral strains [12]. Like social network epidemiology, molecular epidemiology has the potential to identify connections between HCV-infected PWID. Whereas social network epidemiology maps risk pathways between PWID (thereby identifying potential paths of HCV transmission), comparing the genetic make-up of HCV infections of network members using molecular phylogenetics can identify the likely pathways though which HCV has actually been transmitted.

The Networks 2 Study [8], [13], [14] - a longitudinal study that combined molecular and social network epidemiological methods to evaluate HCV transmission dynamics in PWID in Melbourne, Australia – began in 2005. Networks 2 was designed to assess the relationship between social networks of PWID and the molecular phylogenetics of HCV. The investigators previously conducted a cross-sectional networks study but observed only a low level of correlation between social distance (the number of social links defined by recent injecting contacts connecting any two network members) and genetic distance [15]. The low level of association was assumed to be at least partially due to the cross-sectional nature of the study. HCV-infected participants might have been infected for many years, which would explain why the majority of HCV infections detected in the study were genetically unrelated to the isolates obtained from each of the participants' recent injecting partners. Subsequent studies have either been limited by small sample size [16], or lack of relevant social network information [17], [18]. It was hypothesised that a high level of association between the social-injecting network and HCV phylogeny would be observed in the Networks 2 study, given its longitudinal design.

## Materials and Methods

### Ethics Statement

The study conformed to the ethical guidelines of the 1975 Declaration of Helsinki; ethical approval was obtained from the Victorian Department of Health Human Research Ethics Committee (project 02/05). Participation was voluntary, written informed consent was obtained from each participant, and all were offered pre- and post-test counselling for HCV, HBV and HIV.

### Recruitment

Networks 2 is a cohort study of PWID recruited from three major illicit drug markets located across metropolitan Melbourne, Australia. Recruitment utilized a social networks approach: at specified interviews, participants were asked to describe their relationships with up to five injecting partners and to introduce them to our field researchers [13]. Participants were bled and interviewed about their risk behaviour and injecting partners at approximately three-month intervals. Most participants were recruited into the study between July 2005 and January 2006, although recruitment of existing participants' injecting partners continued. This paper reports on data collected between July 2005 and August 2008.

### Laboratory methods

Blood samples were screened for antibodies to HCV (anti-HCV) by a third-generation enzyme immunoassay (Abbott Laboratories, Chicago, IL, USA) and anti-HCV positive specimens were tested again by Murex anti-HCV version 4.0 (Murex Biotech, Kyalami, South Africa) for confirmation. Irrespective of anti-HCV status, all samples were tested for HCV RNA by the COBAS AMPLICOR HCV test version 2.0 (Roche Molecular Systems, Branchburg, NJ, USA). HIV and hepatitis B virus (HBV) status were determined by measuring serological markers as described previously [14].

HCV RNA positive blood samples were genotyped by a reverse-phase hybridisation line probe assay (LiPA, Versant HCV Genotype Assay, Siemens Healthcare Diagnostics, Tarrytown, NY, USA) [19]. For molecular studies, amplification was performed using a nested PCR with primers specific to the core region (331 nucleotides; positions 373–703 relative to the H77 reference sequence [20]) as previously described [21]. Direct sequencing was then performed on the PCR product using ABI PRISM™ Dye Terminator Cycle Sequencing Ready Reaction Kit (PE Applied Biosystems, Foster City, CA, USA) according to the manufacturers' instructions. Sequences with ambiguities (35 of 563) and sequences that were shorter than 331 nucleotides (6 of 563) were removed prior to analysis.

### Phylogenetic analyses

HCV core sequences were aligned using Clustal W through MEGA version 4.0 and MUSCLE [22]–[24]. The alignments were compared using the AltAVisT web tool and edited by hand where appropriate [25]. An initial evolutionary history was inferred using the neighbour-joining method, with sequence distances calculated using the maximum composite likelihood method [26]. The percentage of replicate trees in which the associated taxa clustered together was calculated using a bootstrap test (1000 replicates, 70% cut-off for defining clusters) [27]. All positions containing gaps and missing data were eliminated from the dataset. Neighbour-Joining trees and bootstrapping analyses were conducted in MEGA version 4.0 [23]. A second evolutionary history was inferred using the maximum likelihood approach, assuming a general time reversible model of evolution with unequal rates among sites (four gamma distributed rates), and a proportion of invariable sites; branch support was determined using bootstrapping (1000 replicates, conducted in MEGA version 5.0 [28], [29]) . The results reported in the main manuscript are based on clusters identified using the neighbour-joining phylogeny (branch support determined using a bootstrap test with a cut-off of 70%); maximum likelihood phylogeny was conducted as part of the sensitivity analysis described below (statistical analysis section) and results are presented in Table S1.

For minor genotypes (<20 infections), clusters were verified by phlyogenetic analysis with reference sequences from the Los Alamos HCV Sequence Database. Reference sequences were identified from the database as follows: all HCV sequences of the relevant HCV genotypes that contained the core region were downloaded and checked for sequencing ambiguities. Sequences with ambiguities were removed. If more than 50 sequences were available, random samples of 50 sequences were chosen using the MS Excel random number generator. Only 11 sequences were available for genotype *6l* and 50 for genotype *6e*, all of which were included in the analysis.

In order to estimate the false discovery rate for identifying possible transmission clusters using the viral region analysed, 300 previously published sequences from the same region of the virus were randomly selected from the Los Alamos HCV Sequence Database and analysed as a control experiment. The random sample was stratified by genotype and included 70 genotype 1a and 3a, 50 genotype 1b, 6a, and 6e, and ten 6l sequences. Among those sequences for which the participant identification code was specified in the sequence database, duplicate sequences from the same participant were removed prior to selecting the sample. Sequence alignment and phylogenetic analysis were undertaken using the methods described above. Sequences that clustered in the phylogenetic analyses were investigated using PubMed. Those that were epidemiologically related (from studies of multiple sequences isolated from the same individual) were discarded.

### Social network construction

In general, social networks encompass a set of nodes (points) and edges (connections). In this case (similar to our previous publication [30]), the nodes were study participants and edges were injecting relationships, defined as participants injecting in the same place and time in the three months before interview. For this analysis two social networks were constructed: in the first, henceforth called the *baseline injecting network*, nodes were participants recruited in the main waves early in the study and the injecting partners they reported in their baseline interviews (July 2005–January 2006), and edges were the injecting relationships reported in those participants' first interviews. In the second network, henceforth called the *flattened injecting network*, nodes were participants recruited up to August 2008, and injecting relationships reported throughout the period of interest were included as edges. Both networks were undirected, meaning that the direction of nomination (whether participant A nominated B or vice versa, or whether both participants nominated each other) was discounted. Images of the social networks were constructed using Ucinet 6 and Netdraw 2 [31], [32].

### HCV infection definitions

1. Participants with *HCV infection at enrolment* were defined by a positive HCV RNA test (HCV RNA limit of detection: 50 IU/mL) at the first study visit (irrespective of anti-HCV status).
2. Among participants without *HCV infection at enrolment*, those with *past HCV infection* were defined by a positive anti-HCV test at the first study visit.
3. Participants with *newly acquired primary HCV infection* were defined by either:
  - A positive HCV RNA test and negative anti-HCV test at the first study visit (indicating very early infection); or
  - A negative HCV RNA test and negative anti-HCV test at their first study visit and a subsequent positive anti-HCV/HCV RNA test during follow-up (HCV seroconversion).
4. Some participants had multiple infections during the study. Participants were defined as having a *newly acquired reinfection* if they:
  - tested HCV RNA negative on two occasions (at least 28 days apart) and subsequently tested HCV RNA positive; or
  - tested HCV RNA positive and subsequently tested HCV RNA negative on one occasion, then tested HCV RNA positive; and
    - at least 28 days had elapsed between the HCV RNA negative test and the subsequent positive test; and
    - the sequence distance between the two HCV RNA positive tests was at least 4% in the core region (331 nucleotides). The methodology used to determine the 4% cut-off is described below (definition 6).
5. Participants were defined as having a *new viral strain* if they did not satisfy the definition of reinfection because they had no intervening negative test but had two consecutive HCV RNA positive tests with sequence distance at least 4% in the core region (331 nucleotides). The methodology used to determine the 4% cut-off is described below (definition 6).
6. All blood tests positive for HCV RNA underwent viral sequencing (HCV core region, 331 nucleotides). Viral sequences were compared pairwise, and the maximum composite likelihood distances were calculated. The mean (SD) distance between viral sequences taken from different participants with the same genotype and subtype was 3.5% (1.3%). The cut-off for defining a new viral strain was set at 4%, equal to approximately three standard deviations (3×1.3%) of the distribution of pairwise differences from viral sequences from different participants with the same genotype and subtype. This method for defining a cut-off was based on the method used by Pham and colleagues [33]. When consecutive sequences from the same participant were compared (including sets with intervening blood tests with no viral sequence – for example, if the blood test was HCV RNA negative), 70% of consecutive sequences were identical (distance = 0%).

### Statistical analysis

To address the study aim (to determine the relationship between social networks and molecular phylogenetics in the context of incident HCV infection), the association between reported injecting partnerships (injecting partner - yes/no) and relationships defined by clustering in the phylogenetic analysis (in the same phylogenetic cluster – yes/no), and the association between social geodesic distance (the smallest number of injecting partnerships connecting two nodes) and HCV core sequence distance (maximum composite likelihood) were measured. An adjusted Jaccard similarity coefficient was used to measure the association between the reported injecting partnerships and relationships defined by clustering in the phylogenetic analysis in the baseline and flattened injecting networks. Tau c rank correlation was used to measure the association between social distance and HCV core sequence distance in the baseline and flattened social networks. To control for confounding between HCV genotype and socio-behavioural characteristics and mixing between participants infected with different HCV genotypes at baseline, this analysis was stratified by genotype and undertaken only amongst participants infected with the major genotypes in the study population, *1a* and *3a* (more information is provided in Appendix S1). The HCV core sequence from each participant's most recent HCV RNA positive test was analysed. The statistical significance of observed Jaccard similarity coefficients and tau c rank correlation coefficients was assessed using the quadratic assignment procedure (QAP) [34]. Adjusted Jaccard similarity coefficients and related QAP analyses were undertaken in Ucinet 6 (12500 permutations) [31]. Tau c rank correlations and related QAP analyses were undertaken in Stata 11 (Lakeway Drive, Texas; 5000 permutations). These methods are discussed in detail in Appendix S2.

In order to assess whether the relationship between the phylogenetic clusters and the social networks was dependent on the methodology used to define the phylogenetic clusters or the social network, the following variations were implemented:

- Defining ties by reporting ever having used a needle/syringe before or after the other participant without sterilisation, rather than having used in the same room as the other participant in the past three months;
- Defining phylogenetic clusters using cut-off for branch support of 80% rather than 70%; and
- Defining phylogenetic clusters based on the maximum likelihood phylogeny rather than the neighbour-joining phylogeny.

For each of these variations, an adjusted Jaccard similarity coefficient was used to measure the association between the reported injecting partnerships and relationships defined by clustering in the phylogenetic analysis in the baseline and flattened injecting networks. The statistical significance of observed Jaccard similarity coefficients was assessed using the quadratic assignment procedure (QAP, 12500 permutations). Adjusted Jaccard similarity coefficients were similar across these variations; results from these sensitivity analyses are reported in Table S1.

To explore the effect of conducting this investigation in the context of a longitudinal rather than cross-sectional study, using logistic regression in Stata 11 we investigated whether incident infections and new viral strains increased the likelihood of being in a phylogenetic cluster compared to other infections. Age, gender, ethnicity, neighbourhood of recruitment, and HCV genotype (*1*, *3* or *6*) were considered as potential confounders. Univariable logistic regression was used to identify candidate predictors for inclusion in the multivariable model. Stepwise backward multiple logistic regression was used to select the final model and its goodness-of-fit was assessed using the Hosmer-Lemeshow test.

Finally, we examined whether *newly-acquired infection* or *new viral strain* might be associated with reporting greater numbers of non-recruited injecting partners, thereby negatively confounding the association between the social network and the HCV phylogeny. The Wilcoxon rank-sum test was used to compare the median number of reported injecting partners, and the median number of reported injecting partners not included in the injecting networks, amongst participants with and without *newly-acquired infection* or *new viral strain*. The Wilcoxon rank-sum test was used because these variables were not normally distributed. Wilcoxon rank-sum tests were undertaken in Stata 11.

## Results

### Participants

Between July 2005 and August 2008, 398 participants were recruited into the study. Four more participants were included in this analysis to maximize the completeness of the social network information because they were nominated as injecting partners before August 2008 but recruited at a later date. Of the 402 participants included in the flattened injecting network, 307 were recruited prior to February 2006. Another 19 participants recruited later were nominated as injecting partners before February 2006. Therefore, the baseline injecting network consisted of 326 participants. Participant characteristics were very similar in the baseline and flattened injecting networks (Table 1).

**Table 1:** Participant characteristics at study entry in the baseline and flattened injecting networks.

		Baseline Networkn (%)N = 326^a^	Flattened Networkn (%)N = 402^a^
**Age**	Median (IQR)^b^	25.1 (22.2–29.4)	25.6 (22.7–30.4)
**Gender**	Female	107 (33)	133 (33)
	Male	219 (67)	269 (67)
**Ethnicity**	Australian	226 (70)	279 (70)
	Vietnamese	45 (14)	54 (14)
	Other	50 (16)	63 (16)
**Employment**	Unemployed	236 (74)	294 (74)
	Paid work^c^	70 (22)	85 (22)
	Student^d^	8 (2)	8 (2)
	Other^e^	6 (2)	9 (2)
**Accommodation**	Stable^f^	225 (69)	281 (70)
	Unstable^g^	99 (31)	119 (30)
**Duration of injecting (years)**	Median (IQR)	8 (4–11)	8 (5–12)
**Receptive needle sharing ever**	Yes	216 (66)	269 (67)
	No	110 (34)	133 (33)
**Main drug injected in the three months prior to enrolment**	Heroin	226 (70)	278 (70)
	Speed	50 (15)	62 (16)
	Buprenorphine	37 (11)	40 (10)
	Other	12 (4)	20 (5)
**Number of people injected with in the three months prior to enrolment**	Median (IQR)	4 (2–7)	3 (2–6)
**Number of injections in the month prior to enrolment**	Median (IQR)	24 (10–56)	23 (10–56)

*Table notes:*

a Totals may not sum to *n* due to missing data.

b IQR: interquartile range.

c Including full-time, part-time and casual employment.

d Including full-time and part-time students.

e Pensioners, home-duties.

f Including own home, renting, and living with parents.

g Including homeless, squat and boarding.

### HCV infection

**Figure 1:**
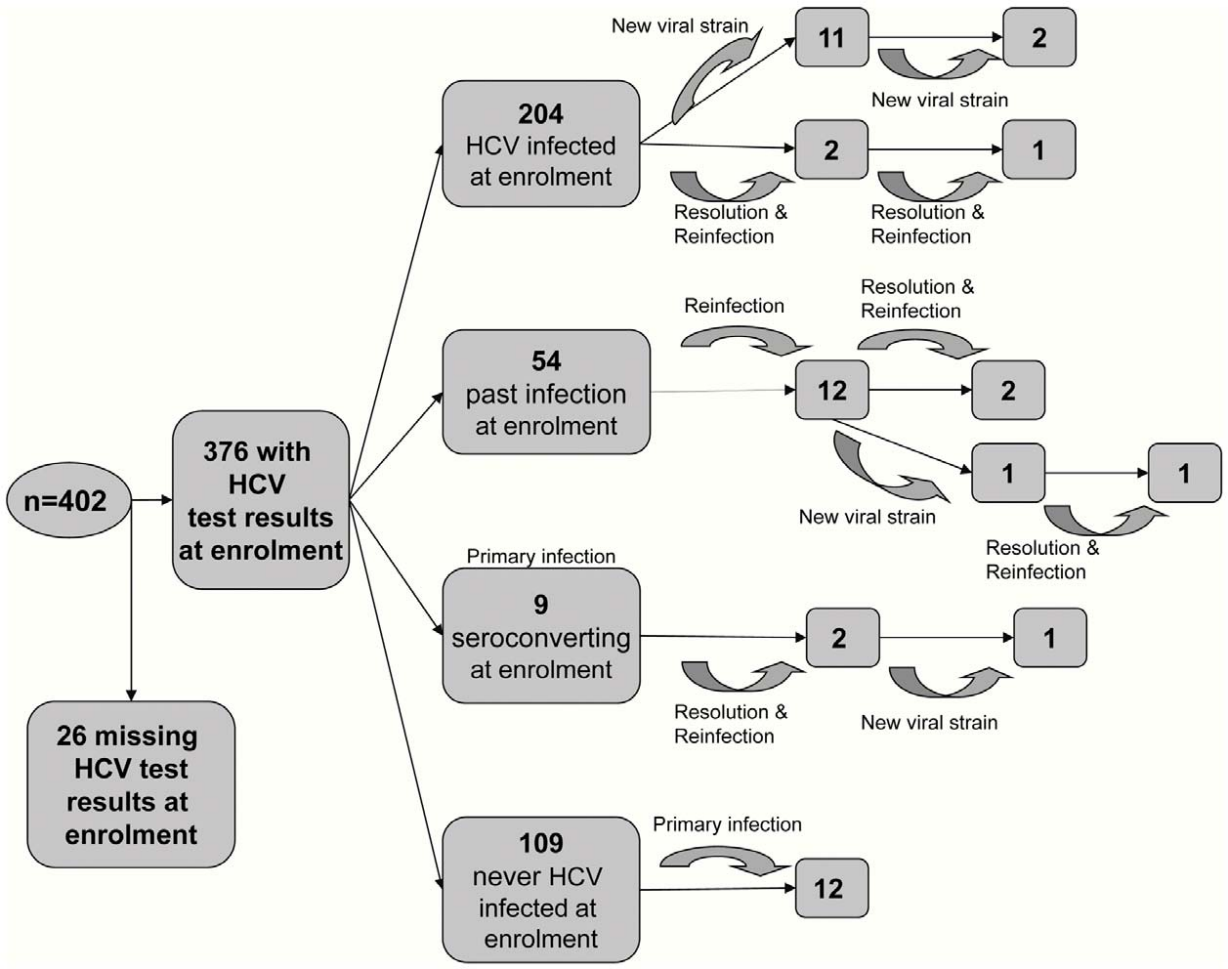
HCV primary infections, reinfections and changes in viral sequence by HCV status at study entry. HCV test results: anti-HCV and qualitative HCV RNA results. Those with missing results could not be bled or had indeterminate results; HCV infection at enrolment: anti-HCV positive and positive HCV RNA test (HCV RNA limit of detection: 50 IU/mL); past HCV infection at enrolment: positive anti-HCV test and negative HCV RNA test at the first study visit; seroconverting at enrolment: anti-HCV negative and positive HCV RNA test at the first study visit (indicating very early infection); never HCV infected at enrolment: negative anti-HCV test and negative HCV RNA test at first study visit. Participants with *newly acquired primary HCV infection* were defined by either: (a) a positive HCV RNA test and negative anti-HCV test at the first study visit (that is, seroconverting at enrolment); or (b) a negative HCV RNA test and negative anti- HCV test at their first study visit (that is, never infected at enrolment) and a subsequent positive anti-HCV/HCV RNA test during follow-up. Participants were defined as having a *newly acquired reinfection* if they tested HCV RNA negative on two occasions (at least 28 days apart) and subsequently tested HCV RNA positive; or tested HCV RNA positive and subsequently tested HCV RNA negative on one occasion, then tested HCV RNA positive, and the sequence distance between the two HCV RNA positive tests was at least 4% in the core region (331 nucleotides). Participants were defined as having a *new viral strain* if they did not satisfy the definition of reinfection because they had no intervening negative test but had two consecutive HCV RNA positive tests with sequence distance at least 4% in the core region (331 nucleotides). The methodology used to determine the 4% cut-off is described in the Materials and Methods section (definition 6).

**Figure 2:**
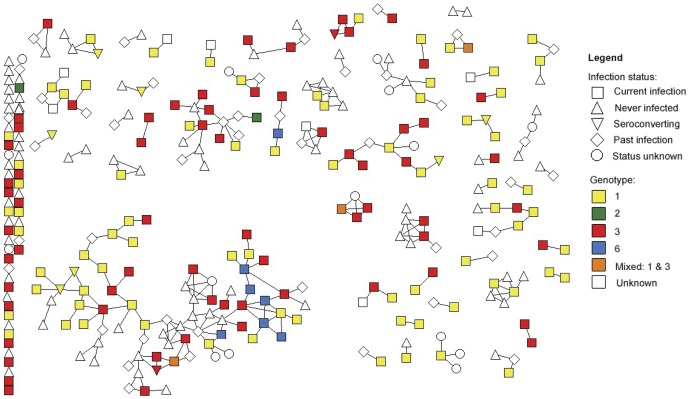
HCV infection status and genotype in the baseline injecting network. Note: The infection status of each participant in the network is denoted by the shape of the node. Amongst participants who were infected at enrolment – that is, infection status is “current infection” or “seroconverting” – genotype is indicated by colour. Nodes that are not coloured represent participants who were not infected at baseline (white upward triangle or diamond) or participants who were infected at baseline but for whom the genotype could not be determined due to insufficient serum and/or low viral load (white square or downward triangle).

Anti-HCV and HCV RNA results were available for 376 participants at study entry. The remaining 26 participants could not be bled or had indeterminate anti-HCV or HCV RNA results at study entry. Initial HCV status and HCV infection events during the study are summarized in Figure 1. In total, 21 newly acquired primary HCV infections and 20 reinfections were observed. Furthermore, in 15 instances participants did not satisfy the definition of reinfection and were defined as having a new viral strain due to substantial change in HCV core sequence (defined in detail above) over two consecutive study visits. The most common genotypes detected were *1* and *3*; amongst participants who were anti-HCV positive at study entry, 45% of infections were genotype *1* and 42% were genotype *3*. Of the newly acquired infections and changes in viral sequence, 39% were genotype *1* and 44% were genotype *3*. Twelve participants were infected with genotype *6* at study entry (6%) and four newly acquired infections were genotype *6* (10%). The distribution of HCV infection and HCV viral genotype in the baseline social network is illustrated in Figure 2.

### HIV and HBV infection

HIV and HBV infection were rare in this cohort. Of the 376 participants with anti-HCV and HCV RNA results at study entry, two (0.5%) were HIV infected – both of these were HCV coinfected – and no additional HIV seroconversions were observed. At study entry, 135 (36%) participants had evidence of prior but not current HBV infection. A further 13 (3%) participants were infected with HBV at study entry (hepatitis B surface antigen positive); eight of these were HCV coinfected, including one who was seroconverting to HCV. Two participants became HBV infected during the study but did not have evidence of HCV coinfection. One of these participants spontaneously cleared their HBV infection; the other became infected with HBV within the last three months of follow-up so it was not possible to determine whether they cleared their infection.

### Injecting networks

The baseline injecting network consisted of 326 participants and 259 injecting relationships (Figure 2). The flattened injecting network consisted of 402 participants and 466 injecting relationships. Participants in the baseline injecting network reported injecting with a median of four people (IQR: 2–7). Throughout the study, participants in the flattened injecting network reported injecting with a median of three other people (IQR: 1–5); typically one of those injecting partners was not recruited into the study (IQR: 0–4). Participants were asked to estimate the duration of each of their reported injecting relationships; the median duration was three years (IQR: 2–6 years). Participants with newly acquired infection (primary or reinfection) or new viral strain reported injecting with similar numbers of people to other participants (median: 3, IQR: 2–4, p = 0.849); and similar to other participants, typically one of their reported injecting partners was not recruited into the study (IQR: 0–2; p = 0.229).

### HCV phlyogeny

**Figure 3:**
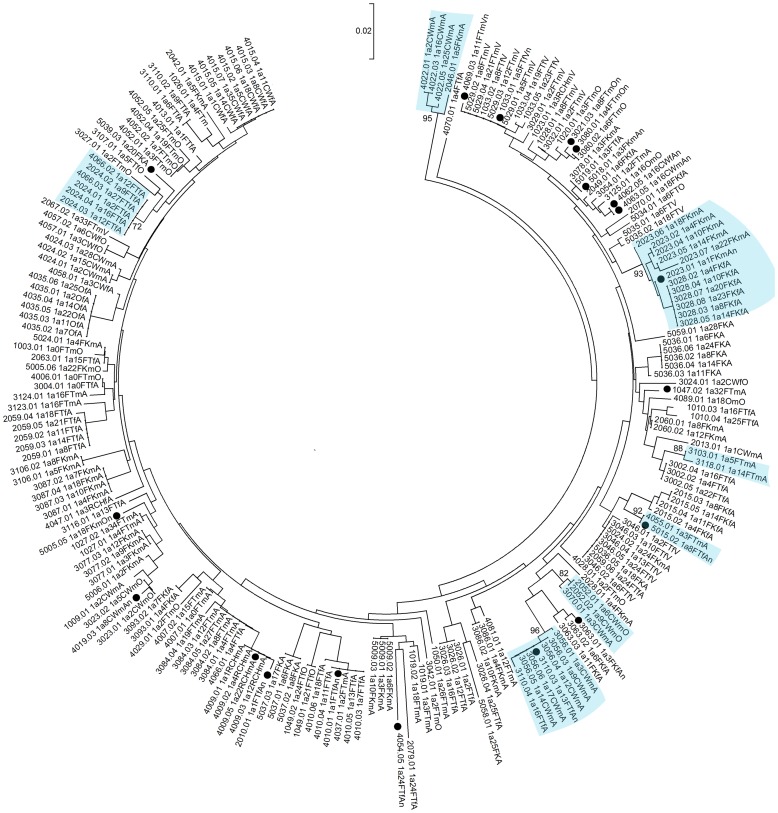
Neighbor-joining analysis of HCV genotype *1a* core sequences. Phylogenetic clusters defined by bootstrap analysis (cut-off 70%) with infections from multiple individuals in the study are highlighted in blue and bootstrap values for these clusters are indicated. Newly acquired infections and changes in viral sequence are denoted using black-filled circles.

**Figure 4:**
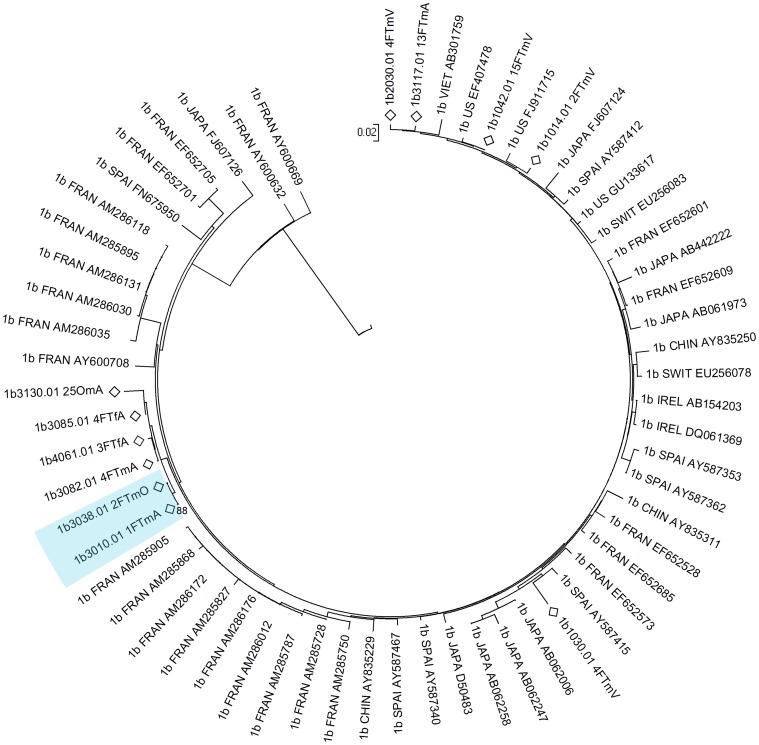
Neighbour-joining analysis of HCV genotype *1b* core sequences. Comparison sequences were genotype 1b sequences randomly selected from the LANL HCV database. Study participants are denoted by diamonds. The first four letters of the name of the country of origin of LANL sequences is included in the ID. Phylogenetic clusters defined by bootstrap analysis (cut-off 70%) with infections from multiple individuals in the study are highlighted in blue and bootstrap values for these clusters are indicated.

**Figure 5:**
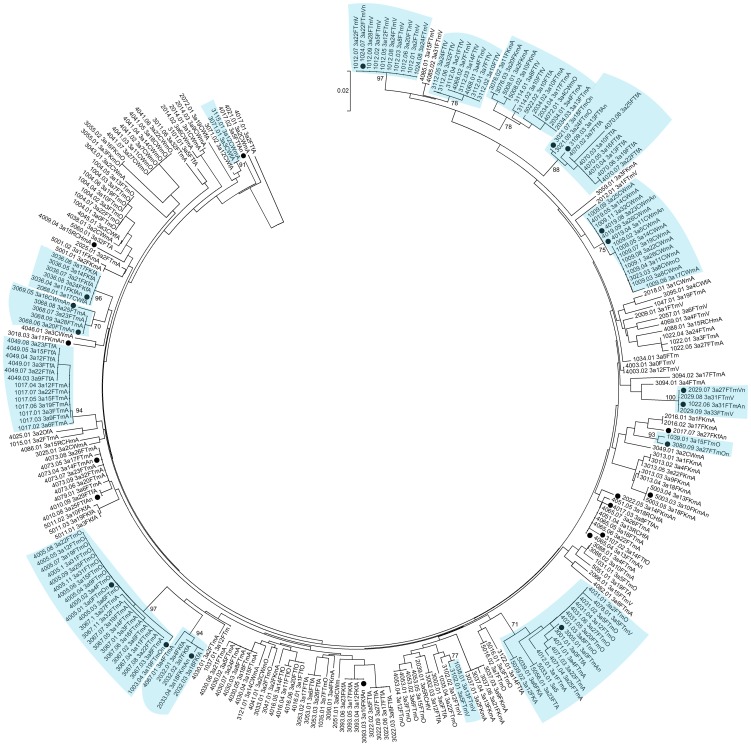
Neighbor-joining analysis of HCV genotype *3a* core sequences. Phylogenetic clusters defined by bootstrap analysis (cut-off 70%) with infections from multiple individuals in the study are highlighted in blue and bootstrap values for these clusters are indicated. Newly acquired infections and changes in viral sequence are denoted using black-filled circles.

**Figure 6:**
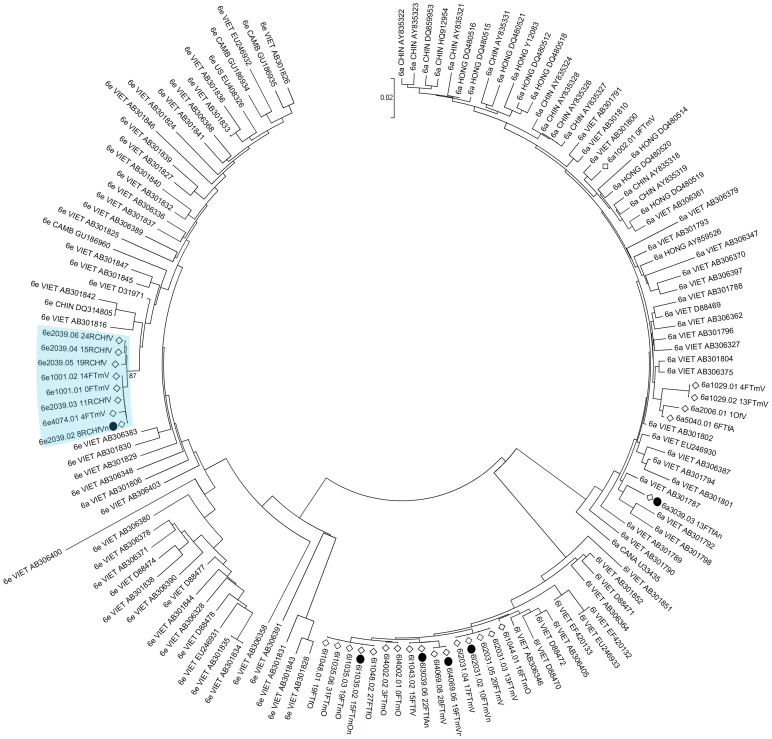
Neighbor-joining analysis of HCV genotype *6a*, *6e*, and *6l* core sequences. Comparison sequences were randomly selected genotype *6a*, *6e*, and *6l* sequences from the LANL HCV database. Study participants are denoted by diamonds. The first four letters of the name of the country of origin of LANL sequences is included in the ID. Phylogenetic clusters defined by bootstrap analysis (cut-off 70%) with infections from multiple individuals in the study are highlighted in blue and bootstrap values for these clusters are indicated. Newly acquired infections and changes in viral sequence are denoted using black-filled circles.

A total of 526 HCV core DNA sequences from 227 study participants (sampled over the course of the study) were obtained from HCV infected participants. Phylogenetic analyses identified 26 clusters containing 69 distinct infections (Figures 3, 4, 5, 6). Within the identified clusters, pairwise nucleotide sequence identity ranged from 98–100%. Phylogenetic clusters incorporated 25% of *HCV infections at enrolment* that were not classified as newly acquired (48 of 195); 48% of *primary infections* (10 of 21), 29% of *reinfections* (5 of 17), and 43% of *new viral strains* (6 of 14). *Newly acquired infections* (primary and reinfection) and *new viral strains* were more than twice as likely to be in phylogenetic clusters than infections present at enrolment that were not classified as newly acquired (OR: 2.07; 95% CI: 1.09–3.94; p = 0.026). This association remained after adjusting for HCV genotype (Table 2; AOR: 2.03; 95% CI: 1.04–3.96; p = 0.037).

**Table 2:** Adjusted and unadjusted odds ratios of an infection being in a phylogenetic cluster.

	Number of infections (%)N = 247	OR (95% CI)	p-value	AOR (95% CI)	p-value
**Incident infection or new viral strain**					
No	195 (79)	1.00			
Yes	52 (21)	2.07 (1.09–3.94)	0.026	2.03 (1.04–3.96)	0.037
**Age group**					
16–22	60 (24)	0.80 (0.39–1.65)	0.543		
23–25	71 (29)	1.26 (0.66–2.39)	0.484		
26+	116 (47)	1.00			
**Gender**					
Male	170 (69)	1.00			
Female	77 (31)	0.87 (0.47–1.59)	0.644		
**Neighbourhood of recruitment**					
Footscray	149 (62)	1.00			
Frankston	46 (19)	0.52 (0.22–1.20)	0.126		
Collingwood/Richmond	46 (19)	1.58 (0.79–3.16)	0.191		
**HCV genotype**					
*1*	114 (46)	1.00			
*3*	116 (47)	2.81 (1.54–5.13)	0.001	2.72 (1.48–4.99)	0.001
*6*	16 (7)	1.02 (0.27–3.91)	0.975	0.87 (0.22–3.40)	0.840
**Ethnicity**					
Australian	161 (66)	1.00			
Vietnamese	40 (16)	1.0 (0.5–2.2)	0.920		
Other	42 (17)	0.8 (0.3–1.7)	0.490		

Note: Goodness of fit for the multivariable model was assessed using the Hosmer-Lemeshow test: p = 0.408.

### Estimated false discovery rate

Among 300 previously published HCV core DNA sequences that were randomly selected from the Los Alamos sequence database, three phylogenetic clusters were supported at the 70% bootstrap level and one was supported at the 80% bootstrap level. These results suggest an approximate false discovery rate of one per 100 infections.

### Associations between social network and phylogenetic clustering patterns

**Figure 7:**
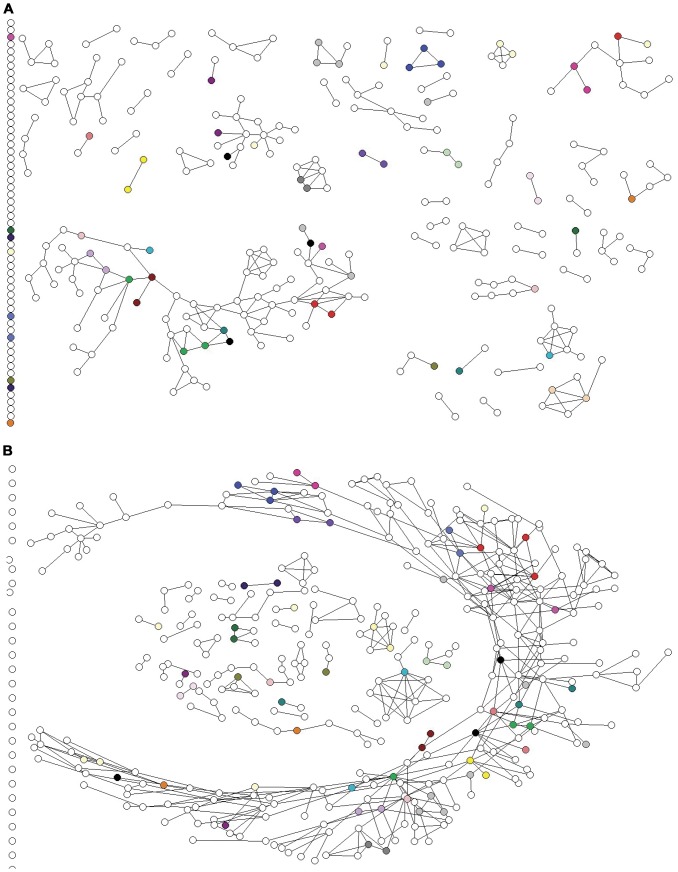
Network diagrams with phylogenetic clusters indicated by colour. Panel A. Baseline injecting network including all injecting ties reported in baseline interviews in main recruitment wave (July 2005–Feb 2006). Phylogenetic clusters identified using data from throughout the study period (July 2005–August 2008). Panel B. Flattened injecting network including all injecting ties reported throughout the study (July 2005–August 2008). Phylogenetic clusters identified using data from throughout the study period (July 2005–August 2008).

Figure 7 illustrates the phylogenetic clusters in the context of the baseline and flattened injecting networks. Of the 67 participants who had sequences in phylogenetic clusters, 32 (48%) were directly connected with at least one other participant in their phylogenetic cluster in the baseline injecting network and 36 (54%) were directly connected with at least one other participant in their phylogenetic cluster in the flattened injecting network. Of the 32 participants directly connected with at least one other participant in their phylogenetic cluster in the baseline injecting network, 22 (69%) reported ever sharing a needle or syringe with that injecting partner and 17 (53%) had done so in the three months before baseline interview. Of the 36 participants directly connected to at least one other participant in their phylogenetic cluster in the flattened injecting network, 30 (83.3%) shared a needle or syringe with that injecting partner during the study period. Of the 20 participants with *newly acquired primary infections or reinfections* or *new viral strains* that were part of phylogenetic clusters, 50% (n = 10) and 65% (n = 13) reported injecting in the same room as other participants in their cluster in the baseline and flattened networks, respectively; 60% (n = 12) reported sharing needles/syringes with other participants in their cluster during the study.

Being in the same phylogenetic cluster was correlated with reported injecting relationships in both the baseline and flattened injecting networks (Table 3). The adjusted Jaccard similarity coefficient was 0.300 for the baseline injecting network and 0.292 for the flattened injecting network. These adjusted Jaccard similarities were highly statistically significant based on the results of the QAP. Indeed, fewer than 0.1% of random permutations in the empirical sampling distribution had adjusted Jaccard similarity coefficients greater than or equal to the observed statistics (p<0.001). These associations were robust to changes in the definition of the social networks (defining networks on the basis of needle/syringe sharing rather than using in the same space), and changes in the definition of the phylogenetic clusters (raising the cut-off for branch support from 70% to 80%, inferring the phylogeny and assessing branch support using a maximum likelihood rather than neighbour-joining approach, or defining clusters on the basis of core sequence identity rather than phylogenetic methods; Table S1).

**Table 3:** Correlation between injecting networks and HCV phylogeny.

Network^1^	Participants^2^	Network measure	Phylogeny measure	Correlation coefficient^3^	p-value^4^	Mean^5^	SD^5^
**Baseline**	*All*	*binary*	*cluster*	*0.300*	*<0.001*	*0.005*	*0.009*
	Genotype *1a* infection	geodesic distance	MCL distance	0.018	0.096	−0.000	0.014
	Genotype *3a* infection	geodesic distance	MCL distance	−0.002	0.419	0.001	0.013
**Flattened**	*All*	*binary*	*cluster*	*0.292*	*<0.001*	*0.006*	*0.009*
	*Genotype 1a infection*	*geodesic distance*	*MCL distance*	*0.071*	*0.017*	*0.000*	*0.034*
	Genotype *3a* infection	geodesic distance	MCL distance	−0.007	0.422	0.001	0.038

Notes:

HCV: hepatitis C virus; MCL: maximum composite likelihood.

1 *Baseline* refers to the baseline injecting network: nodes are participants that were recruited in the main recruitment waves at the beginning of the study; edges are injecting relationships reported in those participants' first interviews. The network is undirected. *Flattened* refers to the flattened injecting network: nodes are participants recruited up to August 2008; edges are injecting relationships reported during this period. The network is undirected.

2 Geodesic distances calculated using complete baseline and flattened networks. Correlations between geodesic distances and MCL distances calculated for the subgroup of participants indicated.

3 Adjusted Jaccard coefficients provided for association between binary injecting networks and phylogenetic clusters. QAP analysis for adjusted Jaccard coefficients conducted in UCINET, 12500 permutations. Tau c coefficients provided for correlation between geodesic injecting network distances and MCL genetic distances. QAP analysis for tau c coefficients conducted in STATA, 5000 permutations. Statistical significance defined as p<0.001. An explanation of the QAP is provided in the materials and methods section.

4 The p-value is based on the percentile of the empirical sampling distribution generated by the QAP in which the observed test statistic falls.

5 The mean and standard deviation of the test statistic in the empirical sampling distribution.

Statistically significant results are presented in *italics*.

In contrast, genetic distance (by maximum composite likelihood) was not well correlated with social distance (geodesic), neither in the baseline social network nor in the flattened social network (Table 3). The Tau c rank correlation coefficients for the baseline and flattened injecting networks respectively were 0.018 and 0.071 for participants with genotype *1a* infection and −0.002 and −0.007 for participants with genotype *3a* infection. The low correlation between genetic distance and social distance (0.072) for genotype 1a infections in the flattened injecting network was considered statistically significant based on the results of the QAP: only 1.7% of the random permutations had tau c coefficients of greater than 0.072 (Table 3). The other correlations were not considered statistically significant based on the results of the QAP: more than 5% of the random permutations had tau c coefficients of greater magnitude than the observed statistic (Table 3).

## Discussion

This study found that participants who had closely related HCV infections (defined as being in a phylogenetically related cluster) were also likely to report having injected together. This is a valuable result because it is an unequivocal empirical demonstration and measurement of the importance of injecting networks in HCV transmission. This has important public health implications due to the considerable opportunities for developing targeted interventions within the injecting social network to prevent HCV transmission (and potentially other infectious diseases). Interestingly, the genetic distance between all studied HCV infections was not well correlated with social distance more broadly, highlighting the complexity of HCV transmission. In addition, the construction of two injecting networks – the baseline injecting network representing a snapshot of the injecting network in the first six months of recruitment, and the flattened injecting network representing injecting relationships reported from 2005–2008 – provides insight into the network epidemiology of HCV transmission in PWID over time.

Injecting network factors have previously been identified as determinants of injecting risk behaviours [7] and HCV infection status [8]–[11], but previous studies of injecting network factors and HCV genetic factors found no association [15]–[17]. Potential reasons included that there were too few infections to detect a statistically significant effect [16], studies lacked relevant injecting contact information [17], or had cross-sectional designs and therefore were limited to comparing recent injecting networks with infections that may have been transmitted years earlier [15]. In contrast to earlier studies, the results of this study establish an association between reported injecting relationships and HCV phylogeny. This shows that the previously identified association between network factors and risk of HCV infection is not simply due to confounding relationships between network factors and HCV risk [8]–[11]. Rather, the study results suggest that the injecting network has a more direct role in HCV transmission that needs to be taken into consideration when developing interventions. Whilst the salience of injecting networks as risk potential networks for HCV transmission may seem intuitive, if injecting partner turnover were high enough or if risk of infection was substantially elevated when injecting with an irregular injecting partner compared to a regular injecting partner, reported injecting relationships may become less relevant [35], [36]. In the context of HCV research, researchers have argued that injecting partner turnover amongst PWID must be high on the basis of data on the average number of injecting partners [35]. However, in this study, even though participants reported multiple concurrent injecting relationships (median number of partners at one time: 3; IQR: 2–5), the median duration of each relationship was also high (3 years; IQR: 2–6 years) relative to duration of injecting career (median: 8 years at baseline: IQR: 5–12). The relatively low reported partner turnover is consistent with the findings that closely genetically related infections were linked to injecting relationships.

Although this study found that those participants with closely related HCV infections were likely to report injecting together, it did not find a strong association between genetic distance and social distance within individual (major) genotypes (*1a* and *3a*). This suggests that a high degree of genetic diversity is already well established in this population within these genotypes and highlights the complexity of HCV transmission and evolution. This observed genetic diversity in the study population is likely to be due in part to the presence of immigrants from many nations among PWID in Melbourne [19]. It is also possible that prison plays a role in the spread of genetically diverse HCV infections. Given the elevated risk of needle-sharing in the prison context, it is possible that PWID inject with new injecting partners and therefore become exposed to HCV strains that they would not encounter outside the prison environment [33], [37]–[39]. We have previously used mathematical modelling to demonstrate how the baseline injecting network would limit HCV transmission [30]; further work is required to model the effect of the social network on viral evolution in more detail.

Given that HCV transmission is fairly uncommon, it is unsurprising that years of injecting network data are required to explain HCV transmission patterns. In this study, the proportion of participants who had sequences in phylogenetic clusters that had direct network connections with at least one of the other participants within that cluster was slightly higher in the flattened social network (53.7%), which represents injecting relationships over the three year study period, than in the baseline injecting network (47.8%), which represents injecting relationships in the first six months of the study. Although numbers were small (n = 20 participants), the difference in proportions was slightly greater amongst participants with newly acquired infection or change in viral sequence, with 50.0% connected to one of the other participants in their phylogenetic cluster in the baseline injecting network and 65.0% in the flattened injecting network. Nonetheless, the level of correlation between HCV phylogeny and injecting network relationships was similar in the baseline and flattened injecting networks. This is because – although more pairs of closely related infections were concordant with reported injecting relationships in the flattened than the baseline injecting network – there were also more reported injecting relationships in the flattened injecting network. In part, the observed association between HCV phylogenetic clusters and injecting partnerships in the baseline injecting network may be attributed to the relatively high number of participants with evidence of newly-acquired primary HCV infection at baseline (anti-HCV negative, HCV RNA positive at study entry). However, as described in the methods section, infections acquired later in the study were also included in the HCV phylogeny that correlated with the baseline injecting network. Therefore it is also possible that this result indicates the presence of relatively long-term injecting relationships that are important to HCV transmission. This is consistent with the self-report data collected on duration of injecting relationships (median: 3 years; IQR: 2–6 years). In this sense, the correlation between HCV phylogeny and the baseline injecting network can be interpreted as indicating that the baseline injecting network is predictive of future HCV transmission throughout the study.

Note that the results of the phylogenetic analysis do not necessarily imply direct transmission pathways. A potential shortcoming of our analyses was the reliance on sequencing of the HCV core gene. This region is relatively conserved so it is possible that some of the phylogenetic clusters are not closely genetically related, but rather distantly related or even unrelated isolates. We found a small amount of phylogenetic clustering in the HCV core gene in sequences randomly selected from the Los Alamos HCV Sequence database. This demonstrates that some of the clustering identified amongst study participant samples might be explained by homoplasy rather than genetic relationships between HCV infections but it is unlikely to explain the larger quantity of phylogenetic clustering identified among study participants (26 clusters identified among study participants compared to a potential three clusters identified among randomly selected previously published sequences). Indeed, in our earlier study of PWID in Melbourne, phylogenetic analyses of the HCV core region were confirmed by analysis of the NS5a region [15]. Therefore, whilst transmission between phylogenetic clusters identified through analysis of the core gene cannot be confirmed without additional analysis of a more heterogeneous region, such as NS5 or the envelope regions, the probability that a pair of unrelated or distantly related sequences would cluster in the HCV phylogeny is likely to be independent of their proximity in the social network, resulting in non-differential misclassification, which reduces the probability of detecting an association but does not confer any risk of detecting a spurious association [40]. In this study, a highly statistically significant association between phylogenetic clustering and the social network was detected; if misclassification of phylogenetic clustering occurred, the true association between genetic relatedness of HCV sequences and the reported social network is likely to be even greater than observed. Note that even where phylogenetic clusters do represent genetically related infections, there may be other genetically related infections or steps in the transmission pathway that were not included in the network or that were not detected because they had spontaneously cleared or were due to superinfection [41]. The correlation between membership in the phylogenetic clusters and reported injecting contacts can therefore be interpreted as indicative of a relationship between genetically closely-related infections (not necessarily direct transmission) and reported injecting partnerships. Notably, HCV detected in this study was transmitted over a long period, with some samples collected in the acute phase of HCV and other participants who may have been infected for a decade or longer. In this context, the relatively conserved core region of the virus provides an opportunity for comparing potentially related isolates that have evolved over a range of timeframes. The core region was also advantageous in that sufficient control sequences were available for the three genotype 6 subtypes circulating in the study population. In the absence of such control sequences, clustering of distantly related sequences from the same subtype is likely to occur [42].

This study has limitations. Data on injecting relationships are based on self-reporting and may be subject to information bias, and relationships could only be included in the analysis if both injecting partners were introduced to study personnel and recruited into the study. This means that in general, the injecting partnerships that were included are likely to represent closer relationships (such as sexual and kinship ties) than those that were not. In the context of HCV transmission, this type of selection bias is likely to result in the inclusion of ties that involve more frequent injection over a longer period of time than other ties and may therefore represent higher than average transmission risk pathways [43]. Nonetheless, and although newly acquired infection was not a predictor of having a large number of unrecruited reported network ties, it is possible that some of the newly acquired infections that were not found to be part of any genetic cluster represent transmission from injecting partners who were not reported or not recruited. In addition, as discussed above, molecular epidemiological analyses were limited to the relatively conserved HCV core region. Finally, it is not valid to use QAP regression for analysis of data collected using snowball sampling; therefore, we were limited to QAP correlation analyses for evaluating the association between injecting network data and HCV phylogeny.

In summary, the finding that participants who had closely related HCV infection (defined by being in a phylogenetic cluster) were likely to also report having injected together is valuable because it demonstrates the importance of the injecting network in HCV transmission. Not only does this highlight the necessity of investigating network factors in studies of HCV transmission, but it raises the possibility of using social network methods in public health interventions aimed at reducing HCV transmission risk. Injecting relationships of the kind reported in this study may be effective pathways for communicating information about safe-injecting practices, and the importance of strong and reasonably lengthy relationships in HCV transmission also raises the idea of developing interventions targeted at injecting partners or groups rather than individuals.

## Supporting Information

Appendix S1 **Correlation between social geodesic distance and genetic distance (maximum composite likelihood) amongst all infected participants regardless of infecting genotype.**
(DOCX)

Appendix S2 **Detailed description of statistical methods.**
(DOCX)

Figure S1 **Distribution of pairwise maximum composite likelihood genetic distance amongst pairs of participants that are and are not connected in the baseline and flattened injecting networks.** Pairs of participants are classified as connected in the social network if there is a path between the two nodes. Baseline refers to the baseline injecting network: nodes are participants that were recruited in the main recruitment waves at the beginning of the study; edges are injecting relationships reported in those participants' first interviews. The network is undirected. Flattened refers to the flattened injecting network: nodes are participants recruited up to August 2008; edges are injecting relationships reported during this period. The network is undirected.
(DOCX)

Table S1 **Sensitivity analysis: effect of social network and infection cluster definitions on the adjusted Jaccard similarity between the social network and clusters of genetically related HCV infections.** HCV: hepatitis C virus. 1. *Baseline* refers to the baseline injecting network: nodes are participants that were recruited in the main recruitment waves at the beginning of the study; edges are injecting relationships reported in those participants' first interviews. The network is undirected. *Flattened* refers to the flattened injecting network: nodes are participants recruited up to August 2008; edges are injecting relationships reported during this period. The network is undirected. 2. The injecting relationship defining the social network. Using refers to reporting using together in the three months prior to interview. Sharing refers to reporting having used a non-sterilised needle/syringe after or before the other person either prior to study entry or during the study period. 3. The method used to define clusters of related infections. NJ: neighbor-joining phylogeny. ML: maximum likelihood phylogeny. 4. Branch support for neighbor-joining was determined using bootstrapping in MEGA 4 (1000 replicates). Branch support for maximum likelihood phylogeny was determined using MEGA 5 (1000 replicates). 5. Adjusted Jaccard coefficients. QAP analysis conducted in UCINET, 12500 permutations. Statistical significance defined as p<0.001. An explanation of the QAP is provided in the materials and methods section. 6. The p-value is based on the percentile of the empirical sampling distribution generated by the QAP in which the observed test statistic falls. 7. The mean and standard deviation of the test statistic in the empirical sampling distribution. All results presented in this table were statistically significant.
(DOCX)

Table S2 **Correlation between injecting networks and HCV phylogeny where all genotypes are considered together and excluding genotype 6.** HCV: hepatitis C virus; MCL: maximum composite likelihood. 1. Baseline refers to the baseline injecting network: nodes are participants that were recruited in the main recruitment waves at the beginning of the study; edges are injecting relationships reported in those participants' first interviews. The network is undirected. Flattened refers to the flattened injecting network: nodes are participants recruited up to August 2008; edges are injecting relationships reported during this period. The network is undirected. 2. Geodesic distances calculated using complete baseline and flattened networks. Correlations between geodesic distances and MCL distances calculated for the subgroup of participants indicated. 3. The p-value is based on the percentile of the empirical sampling distribution generated by the QAP in which the observed test statistic falls. 4. The mean and standard deviation of the test statistic in the empirical sampling distribution. 5. Statistically significant results are presented in italics.
(DOCX)

Table S3 **Spearman rank correlations between injecting networks and HCV phylogeny.** HCV: hepatitis C virus; MCL: maximum composite likelihood. 1. Baseline refers to the baseline injecting network: nodes are participants that were recruited in the main recruitment waves at the beginning of the study; edges are injecting relationships reported in those participants' first interviews. The network is undirected. Flattened refers to the flattened injecting network: nodes are participants recruited up to August 2008; edges are injecting relationships reported during this period. The network is undirected. 2. Geodesic distances calculated using complete baseline and flattened networks. Correlations between geodesic distances and MCL distances calculated for the subgroup of participants indicated. 3. The p-value is based on the percentile of the empirical sampling distribution generated by the QAP in which the observed test statistic falls. 4. The mean and standard deviation of the test statistic in the empirical sampling distribution. 5. Statistically significant results are presented in italics.
(DOCX)

## References

[1] World Health Organization (1999) Global surveillance and control of hepatitis C. J Viral Hepat 6: 35–47. 10847128

[2] Seeff LB (2009) The history of the “natural history” of hepatitis C (1968–2009). Liver Int 29: 89–99. 10.1111/j.1478-3231.2008.01927.x PMC4373556 19207971

[3] Shepard CW, Finelli L, Alter MJ (2005) Global epidemiology of hepatitis C virus infection. Lancet Infect Dis 5: 558–567. 16122679 10.1016/S1473-3099(05)70216-4

[4] Nelson PK, Mathers BM, Cowie B, Hagan H, Des Jarlais D, et al (2011) Global epidemiology of hepatitis B and hepatitis C in people who inject drugs: results of systematic reviews. Lancet 378: 571–583. 21802134 10.1016/S0140-6736(11)61097-0 PMC3285467

[5] Aceijas C, Rhodes T (2007) Global estimates of prevalence of HCV infection among injecting drug users. International Journal of Drug Policy 18: 352–358. 17854722 10.1016/j.drugpo.2007.04.004

[6] Friedman S, Aral S (2001) Social networks, risk-potential networks, health, and disease. J Urban Health 78: 411–417. 11564845 10.1093/jurban/78.3.411 PMC3455917

[7] De P, Cox J, Boivin J-F, Platt RW, Jolly AM (2007) The importance of social networks in their association to drug equipment sharing among injection drug users: a review. Addiction 102: 1730–1739. 17935581 10.1111/j.1360-0443.2007.01936.x

[8] Aitken CK, Lewis JA, Hocking JS, Bowden DS, Hellard ME (2009) Does Information about IDUs' Injecting Networks Predict Exposure to the Hepatitis C Virus? Hepat Mon 9: 17–32.

[9] Latkin C, Yang C, Srikrishnan A, Solomon S, Mehta S, et al (2011) The relationship between social network factors, HIV, and Hepatitis C among injection drug users in Chennai, India. Drug Alcohol Depend 117: 50–54. 21315523 10.1016/j.drugalcdep.2011.01.005 PMC3112240

[10] Wylie JL, Shah L, Jolly A (2007) Incorporating geographic settings into a social network analysis of injection drug use and bloodborne pathogen prevalence. Health & Place 13: 617–628. 17074527 10.1016/j.healthplace.2006.09.002

[11] Wylie J, Shah L, Jolly A (2006) Demographic, risk behaviour and personal network variables associated with prevalent hepatitis C, hepatitis B, and HIV infection in injection drug users in Winnipeg, Canada. BMC Public Health 6: 229. 16970811 10.1186/1471-2458-6-229 PMC1586015

[12] Stumpf MPH, Pybus OG (2002) Genetic diversity and models of viral evolution for the hepatitis C virus. FEMS Microbiol Lett 214: 143–152. 12351222 10.1111/j.1574-6968.2002.tb11338.x

[13] Aitken CK, Lewis J, Tracy SL, Spelman T, Bowden DS, et al (2008) High incidence of hepatitis C virus reinfection in a cohort of injecting drug users. Hepatology 48: 1746–1752. 18844233 10.1002/hep.22534

[14] Miller ER, Hellard ME, Bowden S, Bharadwaj M, Aitken CK (2009) Markers and risk factors for HCV, HBV and HIV in a network of injecting drug users in Melbourne, Australia. J Infect 58: 375–382. 19328555 10.1016/j.jinf.2009.02.014

[15] Aitken CK, McCaw RF, Bowden DS, Tracy SL, Kelsall JG, et al (2004) Molecular epidemiology of hepatitis C virus in a social network of injection drug users. Journal of Infectious Diseases 190: 1586–1595. 15478062 10.1086/424678

[16] Brewer DD, Hagan H, Sullivan DG, Muth SQ, Hough ES, et al (2006) Social structural and behavioral underpinnings of hyperendemic hepatitis C virus transmission in drug injectors. Journal of Infectious Diseases 194: 764–772. 16941342 10.1086/505585

[17] Pilon R, Leonard L, Kim J, Vallee D, De Rubeis E, et al (2011) Transmission Patterns of HIV and Hepatitis C Virus among Networks of People Who Inject Drugs. PLoS One 6: e22245. 21799802 10.1371/journal.pone.0022245 PMC3140499

[18] Romano CM, de Carvalho-Mello IMVG, Jamal LF, de Melo FL, Iamarino A, et al (2010) Social Networks Shape the Transmission Dynamics of Hepatitis C Virus. PLoS One 5: e11170. 20585651 10.1371/journal.pone.0011170 PMC2890415

[19] McCaw R, Moaven L, Locarnini SA, Bowden DS (1997) Hepatitis C virus genotypes in Australia. J Viral Hepat 4: 351–357. 9310934 10.1046/j.1365-2893.1997.00060.x

[20] Kuiken C, Combet C, Bukh J, Shin-I T, Deleage G, et al (2006) A comprehensive system for consistent numbering of HCV sequences, proteins and epitopes. Hepatology 44: 1355–1361. 17058236 10.1002/hep.21377

[21] Dev AT, McCaw R, Sundararajan V, Bowden S, Sievert W (2002) Southeast Asian patients with chronic hepatitis C: the impact of novel genotypes and race on treatment outcome. Hepatology 36: 1259–1265. 12395338 10.1053/jhep.2002.36781

[22] Higgins D, Thompson J, Gibson T, Thompson JD, Higgins DG, et al (1994) CLUSTAL W: improving the sensitivity of progressive multiple sequence alignment through sequence weighting, position-specific gap penalties and weight matrix choice. Nucleic Acids Res 22: 4673–4680. 7984417 10.1093/nar/22.22.4673 PMC308517

[23] Tamura K, Dudley J, Nei M, Kumar S (2007) MEGA4: Molecular Evolutionary Genetics Analysis (MEGA) software version 4.0. Mol Biol Evol 24. 10.1093/molbev/msm092 17488738

[24] Edgar RC (2004) MUSCLE: multiple sequence alignment with high accuracy and high throughput. Nucleic Acids Res 32: 1792–1797. 15034147 10.1093/nar/gkh340 PMC390337

[25] Morgenstern B, Goel S, Sczyrba A, Dress A (2003) AltAVisT : A WWW tool for comparison of alternative multiple alignments. Bioinformatics 19: 425–426. 12584133 10.1093/bioinformatics/btf882

[26] Saitou N, Nei M (1987) The neighbor-joining method: A new method for reconstructing phylogenetic trees. Mol Biol Evol 4: 406–425. 3447015 10.1093/oxfordjournals.molbev.a040454

[27] Felsenstein J (1985) Confidence limits on phylogenies: An approach using the bootstrap. Evolution 39: 783–791. 28561359 10.1111/j.1558-5646.1985.tb00420.x

[28] Tamura K, Peterson D, Peterson N, Stecher G, Nei M, et al (2011) MEGA5: Molecular Evolutionary Genetics Analysis using Maximum Likelihood, Evolutionary Distance, and Maximum Parsimony Methods. Mol Biol Evol 8: 2731–2739. 10.1093/molbev/msr121 PMC3203626 21546353

[29] Nei M, Kumar S (2000) Molecular Evolution and Phylogenetics. New York: Oxford University Press.

[30] Rolls DA, Daraganova G, Sacks-Davis R, Hellard M, Jenkinson R, et al (2012) Modelling Hepatitis C transmission over a social network of injecting drug users. J Theor Biol 297: 73–87. 22185979 10.1016/j.jtbi.2011.12.008

[31] Borgatti SP, Everett MG, Freeman LC (2002) Ucinet for Windows: Software for Social Network Analysis. Harvard, MA: Analytic Technologies.

[32] Borgatti SP (2002) NetDraw: Graph Visualization Software. Harvard, MA: Analytic Technologies.

[33] Pham ST, Bull RA, Bennett JM, Rawlinson WD, Dore GJ, et al (2010) Frequent multiple hepatitis C virus infections among injection drug users in a prison setting. Hepatology 52: 1564–1572. 21038409 10.1002/hep.23885

[34] Simpson W (2001) The Quadratic Assignment Procedure (QAP). STATA usergroup: 1st North American meeting. Boston, Mass. Available: http://www.stata.com/meeting/1nasug/simpson.pdf. Accessed 2012 Oct 3.

[35] Hahn JA, Wylie D, Dill J, Sanchez MS, Lloyd-Smith JO, et al (2009) Potential impact of vaccination on the hepatitis C virus epidemic in injection drug users. Epidemics 1: 47–57. 20445816 10.1016/j.epidem.2008.10.002 PMC2863120

[36] Volz E, Meyers LA (2007) Susceptible–infected–recovered epidemics in dynamic contact networks. Proceedings of the Royal Society B: Biological Sciences 274: 2925–2934. 17878137 10.1098/rspb.2007.1159 PMC2291166

[37] Jurgens R, Nowak M, Day M (2011) HIV and incarceration: prisons and detention. Journal of the International AIDS Society 14: 26. 21595957 10.1186/1758-2652-14-26 PMC3123257

[38] Pollini RA, Alvelais J, Gallardo M, Vera A, Lozada R, et al (2009) The harm inside: Injection during incarceration among male injection drug users in Tijuana, Mexico. Drug Alcohol Depend 103: 52–58. 19386448 10.1016/j.drugalcdep.2009.03.005 PMC2693031

[39] Wood E, Li K, Small W, Montaner JS, Schechter MT, et al (2005) Recent Incarceration Independently Associated with Syringe Sharing by Injection Drug Users. Public Health Rep 120: 150–156. 15842116 10.1177/003335490512000208 PMC1497693

[40] Hennekens CH, Buring JE (1987) Epidemiology in Medicine; Mayrent SL, editor. Philadelphia: Lippincott Williams & Wilkins.

[41] Bernard EJ, Azad Y, Vandamme AM, Weait M, Geretti AM (2007) HIV forensics: pitfalls and acceptable standards in the use of phylogenetic analysis as evidence in criminal investigations of HIV transmission*. HIV Med 8: 382–387. 17661846 10.1111/j.1468-1293.2007.00486.x

[42] Smith DB, Simmonds P (1997) Review: Molecular epidemiology of hepatitis C virus. J Gastroenterol Hepatol 12: 522–527. 9257244 10.1111/j.1440-1746.1997.tb00477.x

[43] Valente T, Vlahov D (2001) Selective risk taking among needle exchange participants: implications for supplemental interventions. Am J Public Health 91: 406–411. 11236405 10.2105/ajph.91.3.406 PMC1446573

