# Supplementary material for: Hepatitis C Virus Phylogenetic Clustering Is Associated with the Social-Injecting Network in a Cohort of People Who Inject Drugs
Source: PLoS One. 2012 Oct 26;7(10):e47335. doi: 10.1371/journal.pone.0047335 (PMC3482197; doi:10.1371/journal.pone.0047335)
Supplement: Appendix S1 — Correlation between social geodesic distance and genetic distance (maximum composite likelihood) amongst all infected participants regardless of infecting genotype. (DOCX) [file pone.0047335.s001.docx]

**Appendix S1**

As noted in the main text of the manuscript, in order to control for confounding between HCV genotype and socio-behavioural characteristics and mixing between participants infected with different HCV genotypes at baseline, correlation analyses between HCV phylogeny and social geodesic distance was stratified by genotype and undertaken only amongst participants infected with the major genotypes in the study population, that is,1a and 3a. In this supplement, the effect of confounding between HCV genotype and socio-behavioural characteristics on the crude (unstratified) correlation between HCV phylogeny and social geodesic distance is illustrated.

Consider Figure S1, the distribution of pairwise genetic distance amongst pairs of participants that are and are not connected in the baseline and flattened injecting networks. Visual inspection shows that the main difference between the distribution of genetic distance amongst connected and disconnected pairs of nodes in both networks is that connected pairs are more likely to have extreme values of genetic distance than disconnected pairs (either very large – greater than 0.2 – or very small – close to 0). The preponderance of very small values of genetic distance amongst connected pairs can be explained by viral transmission. The predominance of very large values of genetic distance amongst connected pairs is surprising but can be explained by confounding between social connectedness and infection with genotype 6 HCV.

A sizeable minority of participants in the Networks 2 study were infected with genotype 6 (5.9% of infections at study entry and 9.8% of newly acquired infections or changes in viral sequence were genotype 6). Genotype 6 is more distant (genetically) to the other genotypes infecting the study population (genotypes 1 and 3) than they are from each other. The mean pairwise genetic distance (maximum composite likelihood) amongst pairs of study participants where one member of the pair is infected with genotype 1 and the other is infected with genotype 3 is 0.17, whereas the mean pairwise genetic distance (maximum composite likelihood) amongst pairs of participants where one member of the pair is infected with genotype 6 and the other is infected with either genotype 1 or genotype 3 is 0.20. However, participants with genotype 6 infection tend to be more socially connected than other participants (see main text, Figure 2). These participants tend to be immigrants from South-East Asia (where genotype 6 is more common) who are reportedly more likely than participants of other ethnicities to be involved in drug dealing, and hence, have more social connections than average [[1](#_ENREF_1),[2](#_ENREF_2)]. This means that there are a considerable proportion of pairs with low social geodesic distance but high genetic distance (because genotype 6 is relatively distant from genotypes 1 and 3).

When participants with genotype 6 infection are included in the analysis, crude correlation between HCV phylogeny and social geodesic distance is small, negative, and statistically significant. However, when participants with genotype 6 infection are removed from the analysis, the correlation between social geodesic distance and genetic distance does not remain statistically significant (Table S2).

**References**

1. Dwyer R, Moore D (2010) Beyond neoclassical economics: Social process, agency and the maintenance of order in an Australian illicit drug marketplace. International Journal of Drug Policy 21: 390-398.

2. Aitken CK, Higgs P, Bowden S (2008) Differences in the social networks of ethnic Vietnamese and non-Vietnamese injecting drug users and their implications for blood-borne virus transmission. Epidemiol Infect 136: 410-416.
