# Supplementary material for: Hepatitis C Virus Phylogenetic Clustering Is Associated with the Social-Injecting Network in a Cohort of People Who Inject Drugs
Source: PLoS One. 2012 Oct 26;7(10):e47335. doi: 10.1371/journal.pone.0047335 (PMC3482197; doi:10.1371/journal.pone.0047335)
Supplement: Appendix S2 — Detailed description of statistical methods. (DOCX) [file pone.0047335.s002.docx]

**Appendix S2**

**Adjusted Jaccard similarity coefficient**

The Jaccard similarity coefficient is a measure of similarity between two binary variables; it was adjusted to account for the asymmetry between the number of reported injecting partnerships and the number of participants who were in the same injecting cluster as other participants. The adjusted coefficient is calculated by taking the quotient of the unadjusted Jaccard similarity coefficient and the maximum possible unadjusted Jaccard similarity coefficient, given the observed number of reported injecting partnerships and the observed number of participants who were in the same phylogenetic cluster.

**Tau c rank correlation**

Tau c rank correlation was used to measure the association between social distance (geodesic) and HCV core sequence distance (maximum composite likelihood). Tau c rank correlation is a non-parametric measure of statistical dependence between two variables, which is suitable for assessing the association between two non-parametric variables such as genetic distance and social distance (geodesic), one of which has ties (in this case, social distance). Tau c rank correlation may be less familiar to some readers than the more commonly used Spearman rank correlation so although *tau c* is more suitable in this case due to ties in the social distance measure, Spearman rank correlations were also assessed and are provided in Table S3.

**The quadratic assignment procedure (QAP)**

The statistical significance of observed Jaccard similarity coefficients and tau c rank correlation coefficients was assessed using the quadratic assignment procedure (QAP) [[27](#_ENREF_27)]. The QAP is used to analyse dyadic data sets where each pair of individuals is considered an observation. Data are represented in matrix form, and each pair of individuals is represented by a valued cell in the matrix. Given that the same individual appears in more than one observation, observations are not independent of one another – i.e., observations within the same row and column are interdependent. The QAP involves permuting the observed data using a random seed to simulate data that correspond to the null hypothesis – that is, data that include no association between HCV phylogeny and the injecting network. Nonetheless, the QAP uses the same permutation algorithm for the rows as for the columns to preserve the dependence structure among observations of the same individual. The permutation procedure is run several thousand times and the test statistic (in this case, the adjusted Jaccard similarity coefficient or tau c correlation coefficient) is calculated for each simulated data set in order to create an empirical sampling distribution. The observed value of the test statistic is considered statistically significant (p-value≤0.05) if it is in the 95^th^ percentile or higher or 5^th^ percentile or lower compared to results from the empirical sampling distribution.
