# Supplementary material for: Hepatitis C Virus Phylogenetic Clustering Is Associated with the Social-Injecting Network in a Cohort of People Who Inject Drugs
Source: PLoS One. 2012 Oct 26;7(10):e47335. doi: 10.1371/journal.pone.0047335 (PMC3482197; doi:10.1371/journal.pone.0047335)
Supplement: Figure S1 — Distribution of pairwise maximum composite likelihood genetic distance amongst pairs of participants that are and are not connected in the baseline and flattened injecting networks. Pairs of participants are classified as connected in the social network if there is a path between the two nodes. Baseline refers to the baseline injecting network: nodes are participants that were recruited in the main recruitment waves at the beginning of the study; edges are injecting relationships reported in those participants' first interviews. The network is undirected. Flattened refers to the flattened injecting network: nodes are participants recruited up to August 2008; edges are injecting relationships reported during this period. The network is undirected. (DOCX) [file pone.0047335.s003.docx]

Figure S1

|  | Flattened network | Baseline network |
| --- | --- | --- |
| Connected |  |  |
| Not connected |  |  |
