# Supplementary material for: Hepatitis C Virus Phylogenetic Clustering Is Associated with the Social-Injecting Network in a Cohort of People Who Inject Drugs
Source: PLoS One. 2012 Oct 26;7(10):e47335. doi: 10.1371/journal.pone.0047335 (PMC3482197; doi:10.1371/journal.pone.0047335)
Supplement: Table S1 — Sensitivity analysis: effect of social network and infection cluster definitions on the adjusted Jaccard similarity between the social network and clusters of genetically related HCV infections. HCV: hepatitis C virus. 1. Baseline refers to the baseline injecting network: nodes are participants that were recruited in the main recruitment waves at the beginning of the study; edges are injecting relationships reported in those participants' first interviews. The network is undirected. Flattened refers to the flattened injecting network: nodes are participants recruited up to August 2008; edges are injecting relationships reported during this period. The network is undirected. 2. The injecting relationship defining the social network. Using refers to reporting using together in the three months prior to interview. Sharing refers to reporting having used a non-sterilised needle/syringe after or before the other person either prior to study entry or during the study period. 3. The method used to define clusters of related infections. NJ: neighbor-joining phylogeny. ML: maximum likelihood phylogeny. 4. Branch support for neighbor-joining was determined using bootstrapping in MEGA 4 (1000 replicates). Branch support for maximum likelihood phylogeny was determined using MEGA 5 (1000 replicates). 5. Adjusted Jaccard coefficients. QAP analysis conducted in UCINET, 12500 permutations. Statistical significance defined as p<0.001. An explanation of the QAP is provided in the materials and methods section. 6. The p-value is based on the percentile of the empirical sampling distribution generated by the QAP in which the observed test statistic falls. 7. The mean and standard deviation of the test statistic in the empirical sampling distribution. All results presented in this table were statistically significant. (DOCX) [file pone.0047335.s004.docx]

**Table S1**

| Network^1^ | Relationship^2^ | Phylogeny^3^ | Branch support cut-off^4^ | Correlation coefficient^5^ | p-value^6^ | Mean^7^ | SD^7^ |
| --- | --- | --- | --- | --- | --- | --- | --- |
| Baseline | using | NJ | 70 | 0.300 | <0.001 | 0.005 | 0.009 |
|  | sharing | NJ | 70 | 0.200 | <0.001 | 0.001 | 0.004 |
|  | using | NJ | 80 | 0.417 | <0.001 | 0.005 | 0.015 |
|  | sharing | NJ | 80 | 0.292 | <0.001 | 0.001 | 0.008 |
|  | using | ML | 70 | 0.243 | <0.001 | 0.005 | 0.009 |
|  | sharing | ML | 70 | 0.186 | <0.001 | 0.002 | 0.005 |
|  | using | ML | 80 | 0.263 | <0.001 | 0.005 | 0.012 |
|  | sharing | ML | 80 | 0.211 | <0.001 | 0.002 | 0.007 |
| Flattened | using | NJ | 70 | 0.292 | <0.001 | 0.006 | 0.009 |
|  | sharing | NJ | 70 | 0.236 | <0.001 | 0.002 | 0.005 |
|  | using | NJ | 80 | 0.379 | <0.001 | 0.006 | 0.014 |
|  | sharing | NJ | 80 | 0.276 | <0.001 | 0.002 | 0.008 |
|  | using | ML | 70 | 0.214 | <0.001 | 0.006 | 0.009 |
|  | sharing | ML | 70 | 0.179 | <0.001 | 0.002 | 0.005 |
|  | using | ML | 80 | 0.212 | <0.001 | 0.006 | 0.011 |
|  | sharing | ML | 80 | 0.173 | <0.001 | 0.002 | 0.006 |
