# Supplementary material for: Hepatitis C Virus Phylogenetic Clustering Is Associated with the Social-Injecting Network in a Cohort of People Who Inject Drugs
Source: PLoS One. 2012 Oct 26;7(10):e47335. doi: 10.1371/journal.pone.0047335 (PMC3482197; doi:10.1371/journal.pone.0047335)
Supplement: Table S2 — Correlation between injecting networks and HCV phylogeny where all genotypes are considered together and excluding genotype 6. HCV: hepatitis C virus; MCL: maximum composite likelihood. 1. Baseline refers to the baseline injecting network: nodes are participants that were recruited in the main recruitment waves at the beginning of the study; edges are injecting relationships reported in those participants' first interviews. The network is undirected. Flattened refers to the flattened injecting network: nodes are participants recruited up to August 2008; edges are injecting relationships reported during this period. The network is undirected. 2. Geodesic distances calculated using complete baseline and flattened networks. Correlations between geodesic distances and MCL distances calculated for the subgroup of participants indicated. 3. The p-value is based on the percentile of the empirical sampling distribution generated by the QAP in which the observed test statistic falls. 4. The mean and standard deviation of the test statistic in the empirical sampling distribution. 5. Statistically significant results are presented in italics. (DOCX) [file pone.0047335.s005.docx]

Table S2

| Network^1^ | Participants^2^ | Network measure | Phylogeny measure | Tau c correlation coefficient | p-value^3^ | Mean^4^ | SD^5^ |
| --- | --- | --- | --- | --- | --- | --- | --- |
| Baseline |  |  |  |  |  |  |  |
|  | *HCV infected* | *geodesic distance* | *MCL distance* | *-0.016* | *0.021* | *0.000* | *0.008* |
|  | HCV infected excluding genotype 6 | geodesic distance | MCL distance | -0.0060 | 0.448 | 0.000 | 0.044 |
| Flattened |  |  |  |  |  |  |  |
|  | *HCV infected* | *geodesic distance* | *MCL distance* | *-0.046* | *0.005* | *-0.000* | *0.019* |
|  | HCV infected excluding genotype 6 | geodesic distance | MCL distance | -0.028 | 0.124 | -0.001 | 0.023 |
